# Comparison of Perioperative Outcomes of Laparoscopic and Single-Port Robotic Adrenalectomy

**DOI:** 10.3390/medicina62071350

**Published:** 2026-07-13

**Authors:** Dohoe Ku, Young Woo Chang, Da Young Yu, Hye Yoon Lee, Gil Soo Son

**Affiliations:** Department of Surgery, Korea University College of Medicine, Seoul 02841, Republic of Korea; kuthekool@gmail.com (D.K.); sweetydy10@gmail.com (D.Y.Y.); heygemma@korea.ac.kr (H.Y.L.); gsson@korea.ac.kr (G.S.S.)

**Keywords:** adrenalectomy, Cushing’s syndrome, hyperaldosteronism, pheochromocytoma, minimally invasive surgical procedures, robotic surgical procedures

## Abstract

*Background and Objectives*: Laparoscopic adrenalectomy is the standard treatment for adrenal tumors, providing reduced postoperative pain, shorter recovery, and improved cosmetic outcomes compared with open surgery. Robotic adrenalectomy was developed to overcome issues resulting from its restricted instrument mobility and two-dimensional vision by providing three-dimensional visualization, tremor elimination, and enhanced dexterity. The single-port (SP) robotic system further reduces invasiveness and improves ergonomics. In this study, we aimed to compare perioperative outcomes between SP robotic and conventional laparoscopic adrenalectomies performed at a single institution. *Materials and Methods*: This retrospective study included data from 114 consecutive patients who underwent adrenalectomy at Korea University Ansan Hospital between January 2015 and July 2025. Among them, 33 patients underwent robotic adrenalectomy using the da Vinci SP system (SP group), whereas 81 underwent conventional laparoscopic adrenalectomy (laparoscopy group). The preoperative characteristics and perioperative outcomes were compared between the two groups. *Results*: There were no significant differences in the demographic or tumor characteristics between the groups. Operation time did not differ significantly between the groups; however, in patients undergoing right adrenalectomy, the mean operation time was significantly shorter in the SP group than in the laparoscopy group. *Conclusions*: SP robotic adrenalectomy demonstrated safety and perioperative outcomes comparable to those of conventional laparoscopic surgery. In right adrenalectomies, the da Vinci SP system achieved a significantly shorter operation time, suggesting that the enhanced instrument flexibility and stable three-dimensional visualization provide a technical advantage in anatomically challenging conditions.

## 1. Introduction

Recent advances in diagnostic imaging and surgical techniques have increased the detection of incidentally identified adrenal masses and the need for surgical management. Since its first report in 1992, laparoscopic adrenalectomy has been established as the standard minimally invasive approach for adrenal masses, with clear advantages over open surgery [1]. Nevertheless, limitations persist, including two-dimensional visualization, limited instrument range of motion, and reliance on an assistant for camera operation [2,3].

Robotic adrenalectomy, which provides three-dimensional vision, improved dexterity, and enhanced ergonomics, was developed to overcome these drawbacks [4,5]. Since the initial report in 1999, robotic platforms have advanced substantially, with studies supporting their safety and feasibility and suggesting potential benefits, such as lower blood loss, shorter hospitalization, and fewer complications [6,7,8]. These benefits are particularly relevant in technically demanding cases involving large tumors or patients with obesity [7,9].

More recently, the da Vinci single-port (SP) system has facilitated single-incision adrenalectomy and has shown promising cosmetic and functional outcomes [10,11]. Nevertheless, comparative evidence between SP and laparoscopic adrenalectomies, especially regarding perioperative outcomes, remains limited [12,13]. Accordingly, in this study, we aimed to evaluate and compare the perioperative outcomes of SP and laparoscopic adrenalectomy to further evaluate their relative clinical benefits.

## 2. Materials and Methods

### 2.1. Patients

In this study, we analyzed data from the medical records of 114 patients who underwent adrenalectomy at Korea University Ansan Hospital, Republic of Korea, between January 2015 and July 2025. Of these, 33 patients underwent robotic adrenalectomy using the da Vinci SP surgical system (Intuitive Surgical, Sunnyvale, CA, USA) (SP group), whereas the remaining 81 patients underwent conventional laparoscopic adrenalectomy (laparoscopy group). All adrenalectomies were performed by a single surgeon (Y.W.C.) to ensure technique uniformity. The da Vinci SP robotic adrenalectomy program was introduced at our institution in 2022. Following its introduction, the choice of surgical approach was determined based on surgeon recommendation and patient preference after discussion of the available treatment options. Patients who were considered suitable candidates for minimally invasive adrenalectomy were offered both surgical options when technically feasible. Patient clinicopathological data, including information on age, sex, body mass index (BMI), tumor size and location, pathological findings, and total operation time, were collected. The operation time was defined as the time from skin incision to skin closure.

### 2.2. Surgical Procedures

**Figure 1:**
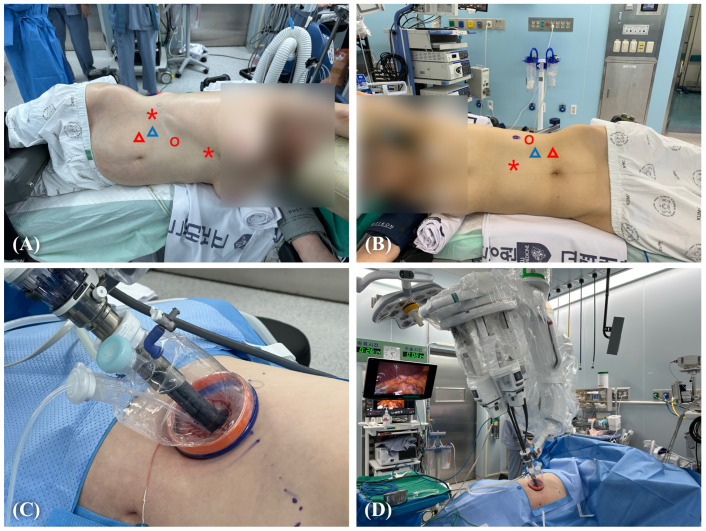
Patient positioning and port placement for laparoscopic and robotic single-port adrenalectomy. (**A**) Patient positioned in the left lateral decubitus position for right adrenalectomy. (**B**) Patient positioned in the right lateral decubitus position for left adrenalectomy. (**C**,**D**) Camera port insertion through a glove port in a patient undergoing single-port adrenalectomy. Red triangle indicates the 12 mm insertion site for laparoscopic camera port; red asterisk indicates 5 mm insertion site for left-hand laparoscopic device and liver retractor; red circle indicates insertion site for right-hand laparoscopic device; blue triangle indicates insertion site for da Vinci SP robotic adrenalectomy. SP, single-port.

Both SP and laparoscopic procedures were performed via the transperitoneal approach, with the patients positioned laterally and the contralateral flank flexed for optimal exposure: left lateral decubitus for right adrenalectomy and right lateral decubitus for left adrenalectomy (Figure 1A and Figure 1B, respectively).

In the laparoscopy group, three ports were used for left-sided procedures and four ports for right-sided procedures. A camera port (12 mm) was placed at the lateral rectus margin at the level of the umbilicus, with 2 additional ports (5 mm for the left hand and 10 mm for the right hand) positioned at the anterior axillary line, 2 cm below the costal margin, and at the epigastric area. For right adrenalectomies, an additional liver retraction port was inserted below the rib for a Nathanson liver retractor.

In the SP group, a 2.0–2.5 cm incision was made along the lateral border of the rectus abdominis above the umbilical line for SP access. A glove port was used for instrument entry, allowing simultaneous insertion of the endoscopic camera and required instruments (Figure 1C,D). The SP cannula was positioned outside the incision, and the custom remote center was aligned at the incision level. Maryland bipolar forceps were placed in arm 1, a monopolar cautery probe in arm 2, and Cadiere forceps in arm 3, maintaining an intra-abdominal pressure of 15 mmHg. A silicone Jackson–Pratt drain, with a diameter of approximately 4.8 mm and trimmed to 2 cm from the suction holes, was inserted for the continuous intraoperative evacuation of smoke and blood, improving visibility and minimizing the need for assistant intervention.

**Figure 2:**
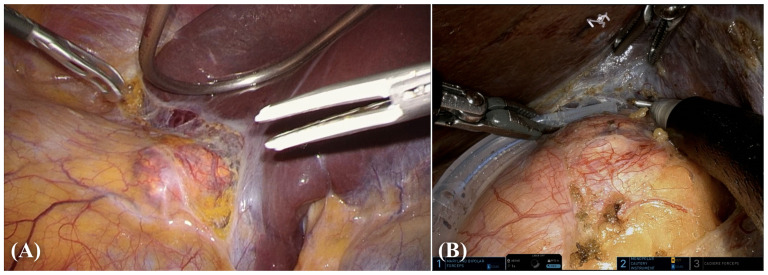
Liver retraction techniques in laparoscopic and robotic single-port adrenalectomy. (**A**) Assisted liver retraction during laparoscopic adrenalectomy. (**B**) Robotic liver retraction during single-port adrenalectomy.

After port placement, adrenalectomy was performed according to the standardized procedure. For left adrenalectomy, the Mattox maneuver was used to mobilize the descending colon and expose the retroperitoneal structures, including the adrenal gland. Once exposed, the adrenal vessels were ligated sequentially, and the gland was dissected from the retroperitoneal bed and retrieved using an endopouch. For right adrenalectomy, exposure was achieved by mobilizing the liver (Figure 2) and dissecting along the lateral border of the inferior vena cava (IVC). The right adrenal vein was carefully isolated and ligated, and dissection was continued until the gland was completely free.

Vascular control was achieved using a robotic Medium-Large Clip Applier (Intuitive Surgical, Sunnyvale, CA, USA) specifically designed for the da Vinci SP system because of the lack of compatible vessel-sealing energy devices. Conventional clips or an energy device were used in laparoscopic adrenalectomy.

### 2.3. Statistical Analyses

All statistical analyses were performed using SPSS software version 25.0 (IBM Corp., Armonk, NY, USA). Continuous variables were compared using Student’s *t*-test, whereas categorical variables were analyzed using the chi-square test or Fisher’s exact test, as appropriate. Results are presented as means ± standard deviations for continuous variables and as numbers (percentages) for categorical variables. Statistical significance was defined as *p* < 0.05.

## 3. Results

### 3.1. Patient Characteristics

The clinicopathological characteristics of the 114 patients included in the study are summarized in Table 1. The mean ages of the patients in the SP and laparoscopy groups were similar (*p* = 0.234), and balanced sex distribution was observed in both groups. No significant difference was observed between the groups in terms of BMI (*p* = 0.715). The tumor size was also comparable across the groups (*p* = 0.264). There was no significant difference in tumor location, with the majority of tumors in both groups occurring on the left side. In addition, no significant differences in clinical diagnoses were observed between the groups (*p* = 0.744), with Cushing’s syndrome being the most frequent diagnosis, followed by pheochromocytoma. The length of postoperative hospital stay also did not differ between the groups (*p* = 0.917). Evaluation of postoperative complications according to the Clavien–Dindo classification revealed no complications above grade II in either group.

### 3.2. Perioperative Parameters

The perioperative parameters of the patients are summarized in Table 2.

**Figure 3:**
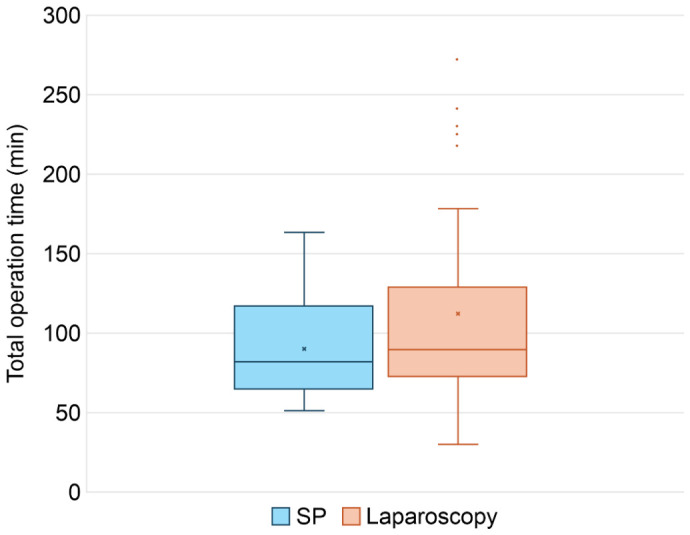
Comparison of overall operation time between the SP and laparoscopy groups. Mean values, coefficients of variation, and ranges are presented. SP, da Vinci single-port system.

The mean operation time did not differ significantly between the groups (*p* = 0.064), despite the smaller coefficient of variation in the SP group compared with the laparoscopy group (Figure 3).

In further analysis, the data were compared according to tumor location (left vs. right). No significant differences in terms of operation time, BMI, or tumor size were observed between the groups in patients undergoing left adrenalectomy. However, the operation time was significantly shorter in the SP group than in the laparoscopy group in patients undergoing right adrenalectomy (*p* = 0.012). BMI and tumor size did not differ significantly between the groups in patients undergoing right adrenalectomy.

## 4. Discussion

Minimally invasive adrenalectomy has become the standard treatment for adrenal tumors, offering advantages such as reduced postoperative pain, shorter postoperative hospital stays, and improved cosmetic and oncological outcomes compared with open surgery [2]. However, despite the wide application of laparoscopic adrenalectomy, issues such as restricted instrument mobility, dependence on two-dimensional visualization, and ergonomic challenges remain, which can make complex dissections technically demanding [4,14,15]. Robotic adrenalectomy was developed to address these shortcomings by offering three-dimensional magnified vision, motion scaling, and tremor filtration [16]. Studies have reported that robotic adrenalectomy provides safety and efficacy comparable to laparoscopic surgery while offering potential ergonomic and technical benefits [17,18].

The SP robotic platform has emerged as an innovative refinement of robotic adrenal surgery [11,17,19]. The da Vinci SP system enables the endoscope and all instruments to enter through a single incision, minimizing port-site trauma and enhancing cosmetic outcomes [20,21]. Its flexible, wristed instruments allow multidirectional movement in confined spaces, which is particularly advantageous in anatomically complex regions [11,22]. Studies have shown that SP robotic adrenalectomy results in safety, feasibility, and perioperative outcomes equivalent to those of multiport robotic approaches [19,23].

In our study, parameters such as BMI, tumor size, and postoperative hospital stay were comparable between the SP and laparoscopy groups, suggesting that the baseline clinicopathological characteristics were well balanced between the two groups. Although the mean operation time did not differ significantly between the groups, the coefficient of variation for operation time was lower in the SP group. Because no formal statistical comparison of the coefficients of variation was performed, this observation should be considered descriptive and interpreted with caution. Previous studies have suggested that features of robotic platforms, including tremor filtration, stable three-dimensional visualization, and improved instrument control, may facilitate a more consistent operative workflow [24,25].

Notably, a significant reduction in the operation time in the SP group compared with the laparoscopy group was observed only in patients undergoing right adrenalectomy. This observation is in agreement with previous reports, which have highlighted the advantages of the robotic system in anatomically challenging cases [6]. Although the right adrenal gland is smaller and anatomically simpler than the left adrenal gland, it lies beneath the liver and immediately adjacent to the IVC, making exposure and vascular dissection technically demanding. In conventional laparoscopy, straight instruments provide limited traction, complicating the separation of the right adrenal gland from the IVC and increasing the risk of retroperitoneal bleeding. In contrast, the da Vinci SP system enables safe and controlled retraction of the right adrenal gland using wristed, articulating instruments with a stable three-dimensional view [11]. The surgeon can also retract the liver directly and without assistance using Cadiere forceps, with the use of a single incision, minimizing the need for extensive liver mobilization. Although these technical advantages may have contributed to the shorter operation time observed during right adrenalectomy in the SP group, because the SP and laparoscopic cohorts were not contemporaneous, the observed reduction in operation time cannot be distinguished from temporal improvements in surgical experience or learning-curve effects. Therefore, this finding should be interpreted with caution and regarded as hypothesis-generating rather than confirmation of an intrinsic advantage of the SP platform.

These findings are consistent with those of the recent comparative study by Akbulut et al., which demonstrated comparable perioperative outcomes between robotic and laparoscopic adrenalectomy despite differences in operation time [26]. While the previous study evaluated conventional multiport robotic adrenalectomy, our study specifically investigated the da Vinci SP platform, thereby providing additional evidence supporting the feasibility and safety of single-port robotic adrenalectomy.

This study has several limitations. First, its retrospective, single-center design and relatively small sample size may have limited the statistical power and restricted the generalizability of the findings. In addition, because the da Vinci SP platform was introduced later during the study period, temporal bias related to accumulated surgical experience, institutional workflow improvements, and perioperative management cannot be completely excluded, despite the comparable baseline clinicopathological characteristics between the two groups. Furthermore, although all procedures were performed by a single experienced endocrine surgeon to minimize inter-surgeon variability, the potential influence of the learning curve associated with the introduction of the da Vinci SP platform cannot be excluded. Future studies evaluating case sequence and cumulative surgical experience will be valuable for better defining the learning curve of the SP platform. Moreover, several clinically relevant perioperative and patient-centered outcomes, including postoperative pain, analgesic use, cosmetic satisfaction, quality of life, and cost-effectiveness, were not consistently available because of the retrospective study design. Prospective multicenter studies with larger cohorts are warranted to validate our findings and further define the clinical benefits of SP robotic adrenalectomy.

## 5. Conclusions

SP robotic adrenalectomy demonstrated perioperative outcomes comparable to those of conventional laparoscopic adrenalectomy. A shorter operation time was observed during right adrenalectomy in the SP group; however, this finding should be interpreted with caution because of the limited sample size. Overall, the da Vinci SP platform appears to be a feasible alternative to conventional laparoscopic adrenalectomy, although further prospective multicenter studies are required to validate these findings.

## Figures and Tables

**Table 1:** Clinicopathological characteristics of patients in the SP and laparoscopy groups.

Characteristic	SP (*n* = 33)	LAP (*n* = 81)	*p*-Value
Age at operation, years	50.2 (25–70)	53.0 (28–76)	0.234
Sex			
Male	16 (48.5%)	39 (48.1%)	0.876
Female	17 (51.5%)	42 (51.6%)	
Body mass index, kg/m^2^	27.8 (21.8–42.8)	26.5 (17.4–48.6)	0.715
Tumor size, cm	3.2 (1.0–7.1)	3.6 (1.0–8.7)	0.264
≤3.0	20 (60.6%)	47 (58.0%)	0.913
>3.0	13 (39.4%)	34 (42.0%)	
Tumor location			0.372
Right	13 (39.4%)	24 (29.6%)	
Left	20 (60.6%)	57 (70.4%)	
Clinical diagnosis			0.744
Nonfunctioning tumor	6 (18.2%)	15 (18.5%)	
Aldosteronism	6 (18.2%)	13 (16.0%)	
Cushing’s syndrome	11 (33.3%)	25 (30.9%)	
Pheochromocytoma	8 (24.2%)	23 (28.4%)	
Malignant	2 (6.1%)	5 (6.2%)	
Postoperative hospital stays, days	3.6 (3–5)	3.6 (2–7)	0.917

Values are presented as mean (range) or number (%). SP, da Vinci single-port system; LAP, laparoscopy.

**Table 2:** Perioperative parameters according to adrenalectomy side and type.

Parameter	Overall			Left			Right		
	SP (*n* = 33)	LAP (*n* = 81)	*p*-Value	SP (*n* = 20)	LAP (*n* = 57)	*p*-Value	SP (*n* = 13)	LAP (*n* = 24)	*p*-Value
Operation time, min	90.5 ± 28.5	104.9 ± 53.0	0.064	99.3 ± 30.0	106.8 ± 57.2	0.461	75.5 ± 18.2	102.0 ± 42.3	0.012
Body mass index, kg/m^2^	27.8 ± 5.2	26.5 ± 4.1	0.242	27.5 ± 5.5	26.9 ± 3.4	0.650	25.9 ± 4.9	26.5 ± 6.0	0.746
Tumor size, cm	3.2 ± 1.4	3.6 ± 1.9	0.219	3.4 ± 1.6	3.8 ± 2.0	0.374	2.9 ± 0.9	3.6 ± 2.0	0.154

Values are presented as mean ± standard deviation. SP, da Vinci single-port system; LAP, laparoscopy.

## Data Availability

The datasets presented in this article are not readily available because of patient privacy and institutional ethical restrictions. Requests to access the datasets should be directed to the corresponding author.

## Abbreviations

The following abbreviations are used in this manuscript:

BMI	Body mass index
IVC	Inferior vena cava
SP	Single port
